# Supplementary material for: The impacts on food purchases and tax revenues of a tax based on Chile’s nutrient profiling model
Source: PLoS One. 2021 Dec 2;16(12):e0260693. doi: 10.1371/journal.pone.0260693 (PMC8638973; doi:10.1371/journal.pone.0260693)
Supplement: S1 Table — (DOCX) [file pone.0260693.s001.docx]

**S1 Table.** **Own, cross price elasticities and income elasticities for food and beverages 2015-2017 (QUAIDS model)**

| **Group** | **Unlabeled beverages** | **Labeled beverages** | **Unlabeled cereal based products** | **Labeled cereal based products** | **Labeled fish and meat** | **Labeled sweet and desserts** |
| --- | --- | --- | --- | --- | --- | --- |
| Price elasticities |  |  |  |  |  |  |
| Unlabeled beverages | -1.052 | 0.080 | 0.028 | -0.086 | -0.011 | -0.027 |
| Labeled beverages | 0.180 | -1.101 | -0.050 | 0.023 | -0.099 | 0.072 |
| Unlabeled cereal based products | 0.138 | 0.003 | -1.086 | 0.187 | -0.060 | 0.090 |
| Labeled cereal based products | -0.152 | 0.048 | 0.149 | -1.212 | 0.126 | -0.009 |
| Labeled fish and meat | -0.006 | -0.202 | -0.121 | 0.167 | -0.813 | -0.021 |
| Labeled sweet and desserts | -0.071 | 0.276 | 0.149 | -0.007 | -0.030 | -1.291 |
|  |  |  |  |  |  |  |
| Income elasticities | 1.067 | 1.074 | 0.728 | 1.050 | 0.996 | 0.974 |

*Own estimations using Kantar data from January 2015 to December 2017. Models adjusted for household size and composition, year, quarter.*
