# Supplementary material for: The impacts on food purchases and tax revenues of a tax based on Chile’s nutrient profiling model
Source: PLoS One. 2021 Dec 2;16(12):e0260693. doi: 10.1371/journal.pone.0260693 (PMC8638973; doi:10.1371/journal.pone.0260693)
Supplement: S2 Table — (DOCX) [file pone.0260693.s002.docx]

**S2 Table. Parameters for revenue estimation.**

| **Parameter** | **Value** | **Source** |
| --- | --- | --- |
| Population |  | https://data.worldbank.org/country/chile |
| 2017 | 18,470,439 |  |
| 2018 | 18,729,160 |  |
| Growth | 1.01% |  |
| Income per capita |  | https://data.worldbank.org/country/chile |
| 2017 | 13,290 |  |
| 2018 | 14,670 |  |
| Growth | 1.10% |  |
| Consumer price index |  | https://www.ine.cl/estadisticas/economia/indices-de-precio-e-inflacion/indice-de-precios-al-consumidor |
| 2017 | 97.34 |  |
| 2019 | 102.26 |  |
| Inflation growth | 1.0614 |  |
|  |  |  |
| Per capita/day volume in grams of ml purchased |  | Kantar 2017 |
| Labeled beverages | 99.4 |  |
| Labeled cereal based products | 17.1 |  |
| Labeled meat and fish | 13.5 |  |
| Labeled sweet and desserts | 13.3 |  |
| Unlabeled beverages | 119.2 |  |
| Unlabeled cereal based products | 44.7 |  |
| Unit values (pesos/ml or gram) |  | Kantar 2017 |
| Labeled beverages | 0.8 |  |
| Labeled cereal based products | 4.0 |  |
| Labeled meat and fish | 3.3 |  |
| Labeled sweet and desserts | 2.6 |  |
| Unlabeled beverages | 0.4 |  |
| Unlabeled cereal based products | 1.2 |  |
