# Supplementary material for: The impacts on food purchases and tax revenues of a tax based on Chile’s nutrient profiling model
Source: PLoS One. 2021 Dec 2;16(12):e0260693. doi: 10.1371/journal.pone.0260693 (PMC8638973; doi:10.1371/journal.pone.0260693)
Supplement: Attachment — Submitted filename: Response to Reviewers.docx [file pone.0260693.s003.docx]

RESPONSE TO KEY POINTS OF THE EDITOR

1. Thank you for clarifying how to access the data Euromonitor data used for this study.

For studies involving third-party data, we encourage authors to share any data specific to their analyses that they can legally distribute. PLOS recognizes, however, that authors may be using third-party data they do not have the rights to share. When third-party data cannot be publicly shared, authors must provide all information necessary for interested researchers to apply to gain access to the data.

a. Please confirm whether future researchers can access the Kantar dataset from Kantar WorldPanel Chile with the contact information provided. Please note that it is not acceptable for this data to be only available upon request to the corresponding author.

**Our Response:** Kantar is a private company. We cannot be sure this same contact will be in place in 2022 or beyond. This person is here now.

the general info is:

Kantar WorldPanel Chile https://[www.kantarworldpanel.com/cl/Sobre-nosotros](http://www.kantarworldpanel.com/cl/Sobre-nosotros) Av. del Valle Nte. 928, Huechuraba

and the info of our contact person is:

María Paz Román email address: [mariapaz.roman@kantar.com](mailto:mariapaz.roman@kantar.com)

b. Please provide a link to the Euromonitor data used in this study within your Data Availability Statement so future researchers can easily access the same data.

Once we have this information we can proceed. We hope to hear from you soon.

**Our Response:**

The linkage on a UNC server set aside for such depository needs is: <https://go.unc.edu/gfrp-data-euromonitor-chile>

2. Thank you for providing the following ethics statement in your submission form:

"This paper uses data approved for exemption by the University of North Carolina’s Institutional Review Board."

Before we proceed, please address the following:

a. Please clarify if the University of North Carolina’s Institutional Review Board approved of this study. If so, please add this information to the ethics statement in your submission form and the ethics statement in your manuscript's methods section. For example, you can state "University of North Carolina’s Institutional Review Board approved of this study."

**Our Response:** Done. Thank you.

b. Please provide more information about participant consent. In the ethics statement in your submission for and the ethics statement in your manuscript's methods section, please ensure that you have specified (1) whether consent was informed and (2) what type you obtained (for instance, written or verbal, and if verbal, how it was documented and witnessed). If your study included minors, state whether you obtained consent from parents or guardians. If the need for consent was waived by the ethics committee, please include this information

**Our Response:** The Kantar survey is completed by heads of household who have provided a written consent. They do this only for heads of households over age 18 for their sample draw which is done randomly.
